# Systematic review and meta-analysis of oral frailty prevalence among older hospitalized patients

**DOI:** 10.3389/fpubh.2025.1681594

**Published:** 2025-12-11

**Authors:** Jie Gong, Liping Tan, Jing Yan, Yuying Qian, Yusu Li

**Affiliations:** Department of Neurosurgery, The Second Affiliated Hospital of Soochow University, Suzhou, China

**Keywords:** hospitalized patients, oral frailty, prevalence, systematic review, meta-analysis

## Abstract

**Background:**

Oral frailty, characterized by reduced oral function, represents a significant yet understudied issue among hospitalized patients, affecting treatment outcomes.

**Objective:**

To determine the prevalence of oral frailty in hospitalized patients and identify high-risk groups through stratified analyses.

**Methods:**

Nine databases, including PubMed, Web of Science, Embase, Scopus, Cochrane Library, CNKI, SinoMed, VIP, and Wanfang, were systematically searched from their inception to July 2025. Data analysis was conducted using Stata 15.0 software, employing a random-effects model to estimate overall prevalence. Subgroup analysis and meta-regression were performed to identify sources of heterogeneity.

**Results:**

This meta-analysis included 27 studies involving 11,575 hospitalized patients. The overall prevalence of oral frailty was found to be 51% (95% CI: 47–55%). Among different disease groups, patients with cancer had the highest prevalence (62, 95% CI: 57–66%), while patients with renal disease had the lowest (39, 95% CI: 32–47%). Regional analysis indicated a prevalence of 52% (95% CI: 48–56%) among patients in mainland China, and 45% (95% CI: 37–53%) in other regions. Cross-sectional studies reported a prevalence of 52% (95% CI: 48–57%), whereas cohort studies reported 42% (95% CI: 36–48%). Disease type accounted for substantial heterogeneity (I^2^ = 94.35%). No publication bias was detected.

**Conclusion:**

The findings suggest that oral frailty is highly prevalent among hospitalized patients, with prevalence varying by disease type. Therefore, establishing specific screening programs for oral frailty and integrating professional oral care into routine medical treatment are recommended to mitigate the risk of complications.

**Systematic review registration:**

https://www.crd.york.ac.uk/PROSPERO/, registration no. CRD420251107503.

## Introduction

Currently, the concept of frailty encompasses multiple dimensions. Researchers have categorized it into physical functions, cognitive capacities, psychological status, and social functions (1). In this context, oral frailty has emerged as a new field, characterized by weakened chewing ability, dysphagia, and decreased tongue pressure. It impairs nutrient intake and exacerbates chronic diseases (2–4).

Hospitalized patients are at high risk for developing oral frailty. The stress of hospitalization may accelerate declining oral function (5). Additionally, acute illness, polypharmacy, and limited self-care capabilities are major contributing factors (6). The systemic inflammatory response triggered by acute conditions directly impairs the reparative function of oral mucosa (7). Clinical statistics indicate approximately 60% of hospitalized patients require medications causing dry-mouth symptoms, averaging over eight medications daily (8, 9). Furthermore, patient mobility restrictions and therapeutic tubes (e.g., nasogastric tubes) may interfere with regular oral hygiene (10, 11). Together, these factors lead to a rapid decline in oral function among hospitalized patients, ultimately resulting in prolonged hospitalization, increased complications, and greater mortality risk (12).

Existing studies have primarily focused on community-dwelling older adults, with limited systematic analysis of hospitalized patients. Consequently, the severity of oral problems in hospitalized settings may be underestimated. Preliminary research data indicate that the prevalence of oral frailty among hospitalized patients ranges from 38 to 67%, significantly higher than the 24–32% prevalence reported for community-dwelling older adults (13–15). Additionally, hospitalized patients face a 1.5–2 times higher risk of complications, such as pneumonia, and experience longer hospital stays compared to community-dwelling older adults (16). Moreover, patients with multiple diseases or prolonged hospitalizations exhibit an increased risk of oral frailty (17). Therefore, more studies on oral frailty among hospitalized patients are necessary to develop preventive and therapeutic measures tailored to hospital settings.

Through a systematic review and meta-analysis, this study synthesizes existing evidence on the prevalence of oral frailty among hospitalized patients. The main objectives are threefold: firstly, to determine the prevalence of oral frailty in hospitalized populations; secondly, to evaluate between-group differences based on disease type, study design, and regional factors; and thirdly, to identify high-risk populations requiring priority interventions. The study’s findings may inform the development of early screening programs and help establish comprehensive oral care systems within hospitals.

## Methods

### Design

This systematic review was registered on PROSPERO, the international prospective register of systematic reviews (registration no. CRD420251107503). The study strictly followed the Preferred Reporting Items for Systematic Reviews and Meta-Analysis (PRISMA) guidelines to ensure scientific rigor and reproducibility (18).

### Selection criteria

Inclusion criteria: (1) cross-sectional or cohort studies; (2) patients hospitalized or requiring regular hospital admissions (e.g., hemodialysis, radiotherapy, chemotherapy); (3) outcome indicator: prevalence of oral frailty; (4) prevalence measured by a validated measurement tool or a reliable, unvalidated tool; and (5) studies published in English or Chinese.

Exclusion criteria: (1) outpatients, patients in nursing or care facilities, patients with dental disease, or patients with a hospital stay of less than 3 days; (2) guidelines, reviews, conference reports, newspapers, and similar documents; (3) duplicate publications; and (4) low-quality literature.

### Search strategy

Nine databases (PubMed, Web of Science, Embase, Scopus, Cochrane Library, CNKI, SinoMed, VIP, and Wanfang) were searched. Search terms combined MeSH terms and free text, including “oral frailty,” “oral weakness,” “oral frail*,” “oral function,” and “patients,” “patient*,” “hospitalize*,” “inpatients,” “inpatient*.” The search spanned from database inception to July 2025. Detailed search strategies are provided in Supplementary Table S1.

### Quality assessment

Cross-sectional studies were evaluated using the Agency for Healthcare Research and Quality (AHRQ) criteria: 8–11 points indicated high quality, 4–7 points moderate quality, and 0–3 points low quality (19). Cohort studies were assessed using the Newcastle-Ottawa Scale (NOS), with scores of 0–3, 4–6, and 7–9 indicating low, medium, and high quality, respectively (20). Evaluations were conducted independently by two researchers, with disagreements resolved by a third reviewer.

### Study selection and data extraction

Two researchers independently screened the literature, extracted data, and cross-checked their findings. A third reviewer assisted in resolving disagreements. Literature was imported into Endnote 21 for preliminary screening by title and abstract. After excluding irrelevant studies, full-text screening was performed according to inclusion and exclusion criteria. Extracted data included author, year, country, study design, sample size, diagnosis, age, prevalence of oral frailty, and assessment tools.

### Data analysis

Statistical analysis was performed using Stata 15.0. Prevalence of oral frailty and 95% confidence intervals (CIs) were calculated. Cochrane’s Q and I^2^ statistics were used to evaluate heterogeneity. A fixed-effects model was applied if I^2^ < 50% and *p* ≥ 0.05. Otherwise, a random-effects model was used. Subgroup analysis and meta-regression were conducted to explore sources of heterogeneity. Funnel plots and Egger’s test were utilized to assess publication bias. Sensitivity analysis was performed using leave-one-out analysis. Statistical significance was set at *p* < 0.05.

## Results

### Search results

**Figure 1:**
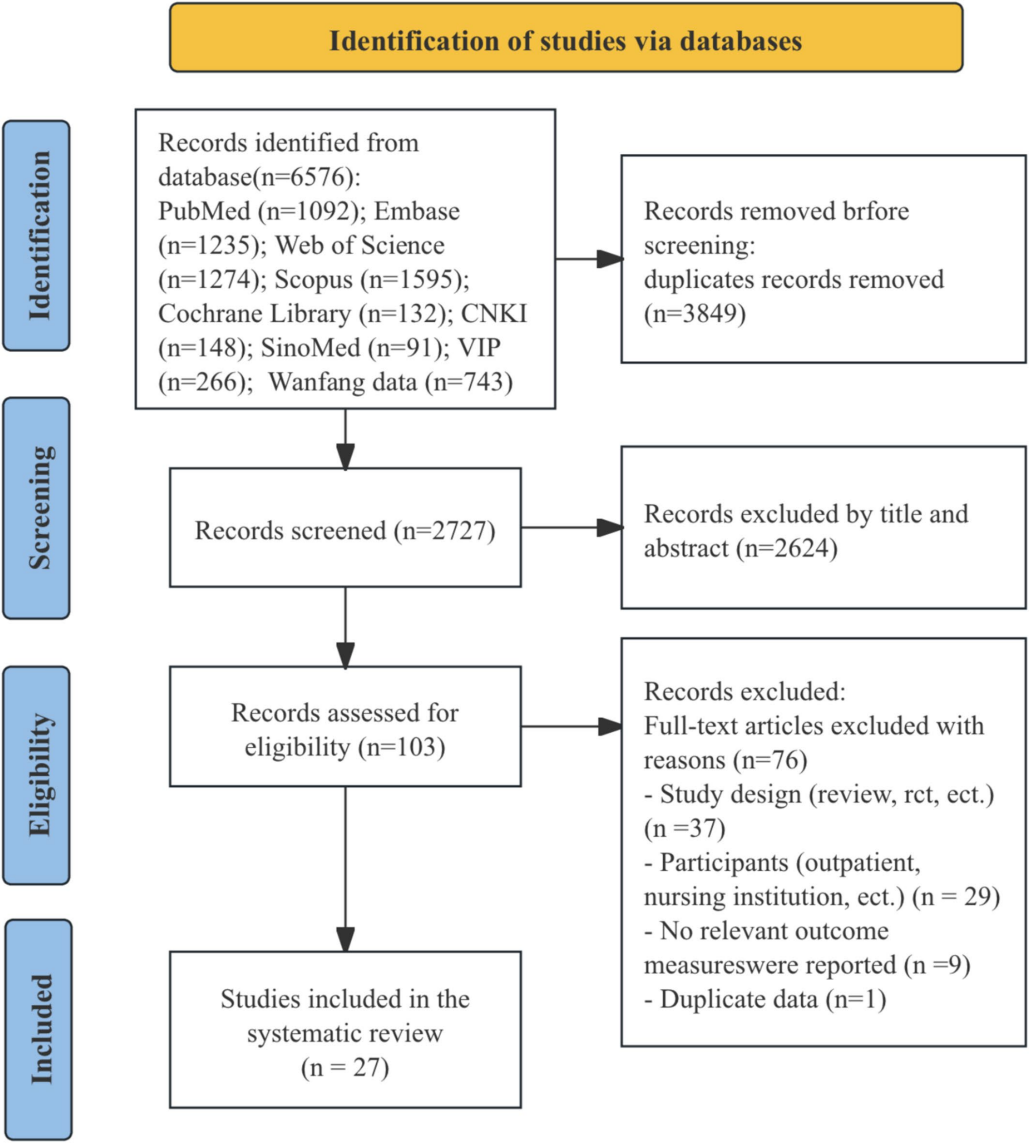
Flow diagram of the study selection process.

The systematic literature search identified 6,576 records. After removing duplicates and screening titles and abstracts, 103 articles underwent full-text review. Of these, 76 studies were excluded: nine lacked relevant outcome measures, 29 did not focus on hospitalized patients, 37 were neither cohort nor cross-sectional studies, and one contained duplicate data. Ultimately, 27 studies were included in the meta-analysis. The selection process is illustrated in Figure 1.

### Study characteristics

The analysis included 27 studies involving 11,575 participants. Most studies adopted cross-sectional designs. Only four used cohort methods. Studies were predominantly conducted in China and Japan and focused on hospitalized adults, especially older adult patients and those with conditions such as hemodialysis, stroke, diabetes, and cancer. Oral frailty prevalence ranged from 28.57 to 67.70%. Almost all studies utilized the Oral Frailty Index-8 for assessment. Detailed study characteristics are summarized in Table 1 and Supplementary Table S2.

**Table 1:** Characteristics of included studies.

Author, Year	Country	Study design	Sample size	Diagnosis	Age	Prevalence of oral frailty (%)	Assessment tools	Quality score
Chen et al. 2024 (38)	China	Cross-sectional	325	Maintenance hemodialysis	≥60	45.23	OFI-8	H
Chen et al. 2023 (39)	Taiwan	Cross-sectional	168	Pneumonia	68.73 ± 12.30	58.30	Modified oral frailty tool	M
Chen et al. 2024 (40)	Taiwan	Cross-sectional	103	>3 days of hospitalization	65.90 ± 11.80	53.40	Modified oral frailty tool	H
Miyasato et al. 2024 (41)	Japan	Prospective cohort	201	Hemodialysis	69.80 *±* 13.20	38.80	OFI-8	H
Xie et al. 2024 (42)	China	Cross-sectional	126	Cerebral small vessel disease	65.43 *±* 8.640	28.57	OFI-8	H
Yang et al. 2024 (43)	China	Cross-sectional	504	Acutely inpatients	77.27 ± 7.67	41.27	OFI-8	H
Dou et al. 2025 (13)	China	Cross-sectional	914	Chronic diseases	≥45	48.70	OFI-8	M
Hu et al. 2024 (44)	China	Prospective cohort	303	Non-cardiac surgery	65–90	49.51	OFI-8	H
Ikuno et al. 2024 (45)	Japan	Retrospective cohort	791	Elective abdominal visceral surgery	≥65	43.74	OFI-8	H
Kobayashi et al. 2025 (46)	Japan	Prospective cohort	51	Peritoneal dialysis	59.1 ± 12.8	29.41	OFI-8	H
Li et al. 2025 (47)	China	Cross-sectional	363	Chemotherapy	≥18	57.58	OFI-8	H
Luo et al. 2025 (48)	China	Cross-sectional	431	T2DM	71.44 ± 7.47	32.95	OFI-8	H
Ma et al. 2025 (49)	China	Cross-sectional	1,115	Stroke	≥60	47.80	OFI-8	H
Tian et al. 2025 (50)	China	Cross-sectional	464	T2DM	≥60	45.90	OFI-8	H
Li et al. 2025 (51)	China	Cross-sectional	852	Hospitalized older adults	≥60	58.20	OFI-8	M
Lu et al. 2025 (52)	China	Cross-sectional	406	Cancer	≥60	63.50	OFI-8	M
Wu et al. 2024 (53)	China	Cross-sectional	388	Chronic diseases	≥60	30.40	OFI-8	M
Shao et al. 2025 (54)	China	Cross-sectional	490	Chronic diseases	≥60	60.20	OFI-8	M
Fan et al. 2024 (55)	China	Cross-sectional	226	Stroke	≥60	61.50	OFI-8	M
Shang et al. 2025 (56)	China	Cross-sectional	220	Diabetes	≥60	46.82	OFI-8	M
Hu et al. 2024 (57)	China	Cross-sectional	379	Hospitalized older adults	≥60	55.40	OFI-8	H
Li et al. 2024 (58)	China	Cross-sectional	546	Hospitalized older adults	≥60	59.20	OFI-8	M
Wang et al. 2025 (59)	China	Cross-sectional	586	Chronic diseases	NR	61.43	OFI-8	H
Shi et al. 2025 (60)	China	Cross-sectional	220	Ischemic stroke	71.84 ± 7.90	58.18	OFI-8	M
Li et al. 2024 (61)	China	Cross-sectional	880	Hospitalized older adults	≥60	58.50	OFI-8	M
Li et al. 2024 (62)	China	Cross-sectional	207	Cancer	63.61 ± 10.48	64.30	OFI-8	H
Wang et al. 2025 (63)	China	Cross-sectional	316	Stroke	≥18	67.70	OFI-8	H

OFI-8, Oral Frailty Index-8; T2DM, type 2 diabetes mellitus.

### Risk of bias

The cohort studies were evaluated using the NOS tool, and all four studies received high-quality ratings. Cross-sectional studies were assessed using the AHRQ tool. Out of 23 studies, 12 were rated as high quality, and the remaining 11 were of moderate quality (Supplementary Tables S3, S4).

### Prevalence of oral frailty

**Figure 2:**
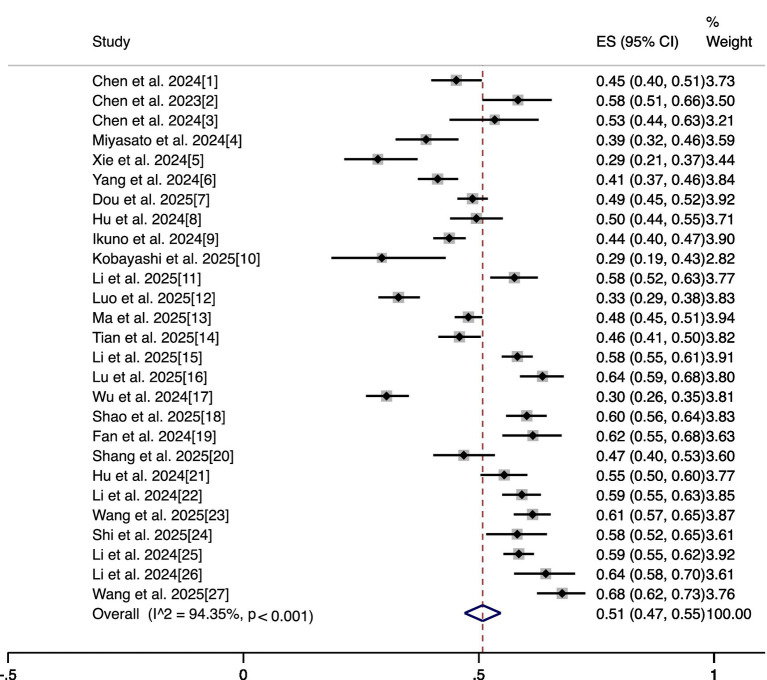
Forest plot showing the prevalence of oral frailty.

The pooled prevalence estimate of oral frailty among hospitalized patients from the 27 studies was 51% (95% CI: 47–55%). Significant heterogeneity existed (I^2^ = 94.35%, *p* < 0.001). These results are presented in Figure 2.

### Subgroup analysis and meta-regression

Subgroup analysis demonstrated significant variations in oral frailty prevalence across different disease types. Patients with cancer had the highest prevalence (62, 95% CI: 57–66%), while patients with renal disease had the lowest (39, 95% CI: 32–47%). Prevalence among patients with chronic diseases was 54% (95% CI: 49–60%), neurological disease patients had a prevalence of 51% (95% CI: 41–61%), and surgical patients 48% (95% CI: 42–53%). Regional comparisons showed that the prevalence in Mainland China (52, 95% CI: 48–56%) was higher than in other regions (45, 95% CI: 37–53%). Study design also influenced the results: cross-sectional studies reported a higher prevalence (52, 95% CI: 48–57%) compared to cohort studies (42, 95% CI: 36–48%). Stratification by age showed that patients aged 60 years and older had a slightly higher prevalence (52, 95% CI: 47–57%) compared to mixed-age groups (50, 95% CI: 43–56%). Additionally, studies with sample sizes of 500 or fewer participants reported a lower prevalence (50, 95% CI: 44–56%) compared to those with larger sample sizes (52, 95% CI: 47–57%). Table 2 details these findings.

**Table 2:** Subgroup analysis for oral frailty prevalence.

Category	No. of studies	Prevalence (95% CI)	I^2^ (%)	*p*-value	Weight (%)
Disease type					
Renal diseases	3	0.39 (0.32–0.47)	66.80	0.05	10.14
Chronic diseases	8	0.54 (0.49–0.60)	94.80	<0.001	34.39
Surgical patients	3	0.48 (0.42–0.53)	62.92	0.07	10.83
Neurological diseases	6	0.51 (0.41–0.61)	95.47	<0.001	22.22
Cancer	3	0.62 (0.57–0.66)	46.00	0.16	11.18
Diabetes	3	0.42 (0.32–0.51)	90.05	<0.001	11.24
Age group					
≥60 years	14	0.52 (0.47–0.57)	94.07	<0.001	53.24
Mixed ages	13	0.50 (0.43–0.56)	94.96	<0.001	46.76
Region					
Mainland China	22	0.52 (0.48–0.56)	94.87	<0.001	82.98
Other regions	5	0.45 (0.37–0.53)	83.95	<0.001	17.02
Study design					
Cross-sectional	23	0.52 (0.48–0.57)	94.64	<0.001	85.98
Cohort	4	0.42 (0.36–0.48)	73.03	0.01	14.02
Sample size					
≤500	19	0.50 (0.44–0.56)	94.66	<0.001	68.85
>500	8	0.52 (0.47–0.57)	94.18	<0.001	31.15
Overall	27	0.51 (0.47–0.55)	94.35	<0.001	100.00

Meta-regression analysis included five predictors: disease type, age group, sample size, study design, and country. Disease type was significantly associated with oral frailty prevalence (*p* = 0.035). Study design showed a marginally significant trend (*p* = 0.066). In contrast, age group, sample size, and country were not significantly associated (*p* > 0.05; Table 3).

**Table 3:** Meta-regression results for oral frailty prevalence.

Predictor	Coefficient (95% CI)	*p*-value	τ^2^	Adj *R*^2^ (%)
Disease type	−0.029 (−0.056, −0.002)	0.035*	0.0099	14.31
Age group	0.023 (−0.068, 0.113)	0.613	0.0119	−3.51
Sample size	−0.022 (−0.120, 0.075)	0.641	0.0120	−3.66
Study design	0.114 (−0.008, 0.235)	0.066†	0.0105	8.84
Country	−0.069 (−0.186, 0.048)	0.236	0.0114	1.58

* *p* < 0.05, † 0.05 ≤ *p* < 0.10.

### Sensitivity analysis and publication bias

**Figure 3:**
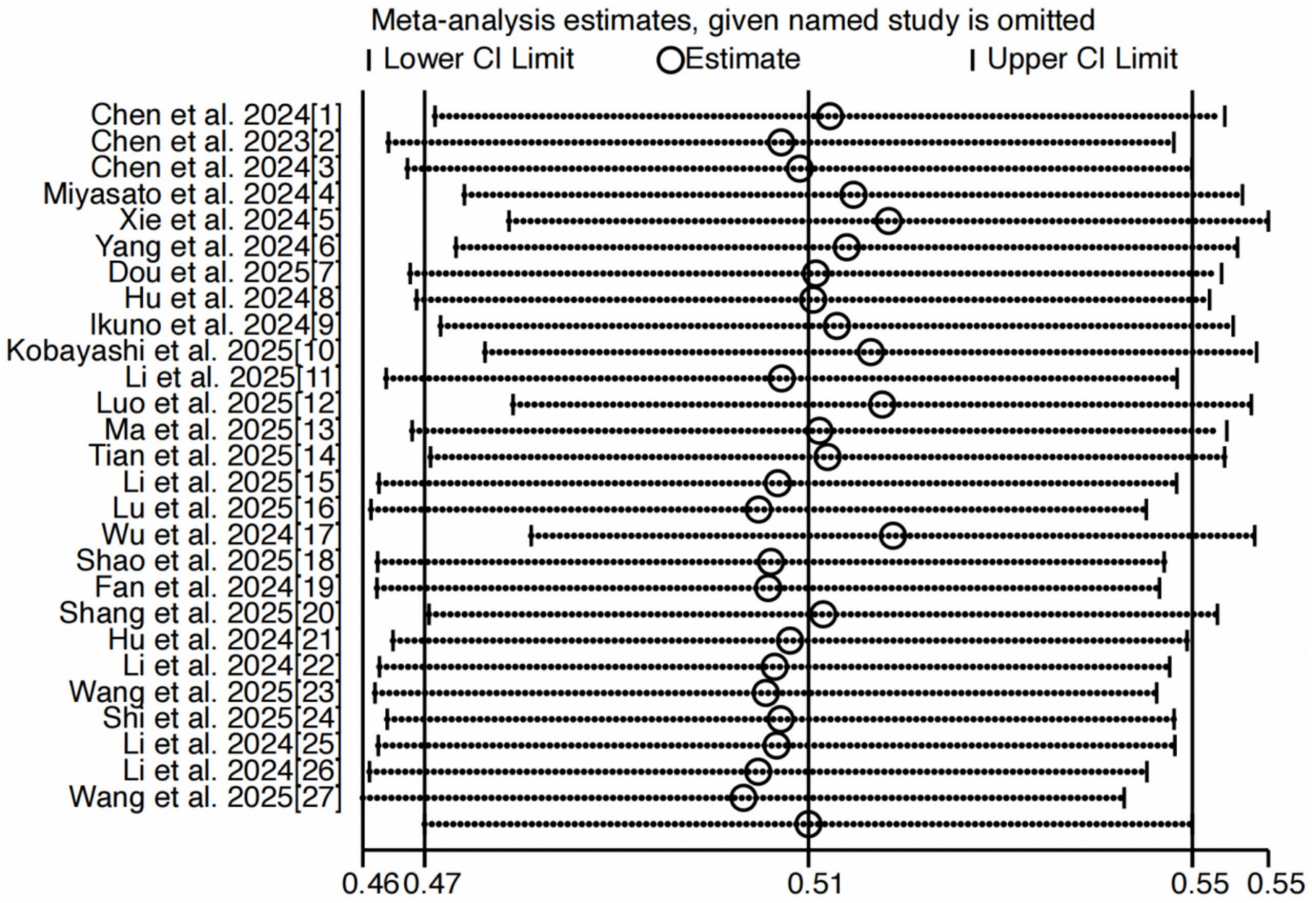
Sensitivity analysis.

**Figure 4:**
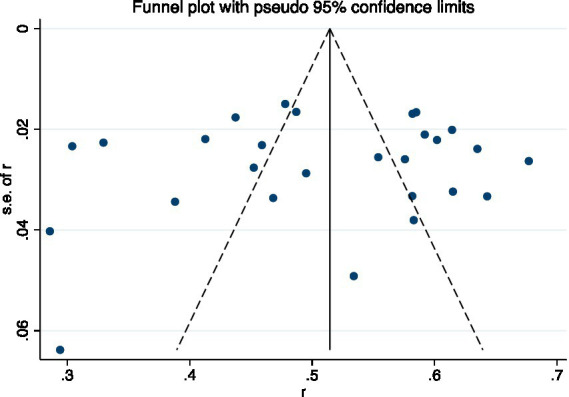
Funnel plot.

Sensitivity analysis demonstrated stable results. When each study was sequentially removed, the overall prevalence of oral frailty remained statistically consistent (Figure 3). Additionally, the funnel plot (Figure 4) and statistical evaluation (Egger’s test: *t* = −0.39, *p* = 0.703; Supplementary Figure S1) showed no substantial publication bias among included studies.

Based on this, a sensitivity analysis was conducted for the cohort studies. Excluding any single study resulted in an estimated prevalence ranging from 0.41 to 0.46, indicating that the overall estimate remains relatively robust (Supplementary Figure S2).

## Discussion

This meta-analysis combined data from 27 studies involving 11,575 hospitalized patients. The results showed that the overall prevalence of oral frailty was 51% (95% CI: 47–55%), significantly higher than the 32% prevalence among community-based older adult populations (21). It was also higher than the 28% reported in Japanese acute care facilities but was consistent with data from Chinese tertiary care hospitals (53%) (21–23). Moreover, oral frailty prevalence was twice as high as somatic (25%) and cognitive (22%) frailty (24). This difference may result from acute impairment of oral function triggered by illness, reduced salivary secretion due to polypharmacy (e.g., anticholinergic agents), and limited self-care for oral hygiene during hospitalization (12). Therefore, a standardized clinical screening process for oral frailty should be established, and oral health assessment should become part of routine admission examinations. Targeted oral health interventions should be implemented during hospitalization, and follow-up monitoring mechanisms should be established post-discharge to prevent further deterioration. Due to significant heterogeneity, meta-regression analysis identified disease type and study design as primary factors influencing prevalence estimates.

Subgroup analyses demonstrated significant differences in oral frailty prevalence across patient groups. Patients with cancer had the highest prevalence (62, 95% CI: 57–66%), approximately 1.6 times higher than patients with kidney disease (39, 95% CI: 32–47%). This disparity may originate from two factors. Pathophysiologically, cancer treatments (e.g., radiotherapy, chemotherapy) frequently lead to oral mucositis and salivary gland damage (25). From a clinical management perspective, patients with renal disease regularly receive oral health monitoring, which might offer protective benefits (26). Differences among surgical patient groups were not significant, suggesting standardized perioperative oral care might temporarily mitigate oral frailty risks. These findings support developing targeted screening strategies for different patient populations. High-risk groups, such as cancer patients, should undergo stricter monitoring criteria.

The prevalence of oral frailty in hospitalized patients in mainland China (52, 95% CI: 48–56%) was significantly higher than that reported in other regions (45, 95% CI: 37–53%). This difference could relate to features of different healthcare systems. Developed countries such as Japan achieve lower prevalence rates through mandatory inpatient dental consultations and community-based preventive programs (27, 28). However, prevalence estimates from China may be overestimated due to study sample bias (81.48% of included studies originated from mainland China). This phenomenon reflects global disparities in healthcare resource allocation. In resource-constrained areas, emergency care is often prioritized over oral health management. Therefore, health systems integrating equity and effectiveness of oral care interventions are necessary.

Study design significantly influenced the estimated prevalence of oral frailty. The prevalence in cross-sectional studies was 52%, 10 percentage points higher than in cohort studies (42%). First, cross-sectional studies included broader patient populations, often encompassing emergency and critically ill cases, while cohort studies typically followed patients in more stable conditions. Second, although both study types record baseline data, cross-sectional studies assess patients predominantly during acute illness phases. Cohort studies might miss periods of critical functional changes due to follow-up intervals (29, 30). Future studies should adopt uniform enrollment criteria, standardized assessment tools, and repeated measurements at defined time points to improve result comparability.

The prevalence of oral frailty among patients aged ≥60 years was 52%, slightly higher than the 50% found in mixed-age group. However, this small difference (2%) was significantly less than the variability (23%) seen across disease types (e.g., cancer versus kidney disease patients). Thus, age alone appears to have limited predictive value for oral frailty (31). In contrast, disease type and related factors exert more significant influences. Hospital-based oral health screening programs should prioritize high-risk patient groups rather than age-based criteria alone.

High heterogeneity was observed in this study. Subgroup analysis identified some factors contributing to this heterogeneity, but additional factors warrant investigation. The predominant tool utilized in most studies included in our research was OFI-8. However, it is imperative to assess the cross-cultural validity of OFI-8, particularly across different language versions such as Chinese, Japanese, and local adaptations, as these variations may impact the overall prevalence estimate. Discrepancies in cut-off values utilized among different studies, influenced by cultural and linguistic factors, pose challenges for result comparison (32). Therefore, future research should meticulously evaluate the performance of OFI-8 versions across diverse cultures and develop and validate tools tailored to specific cultural contexts to enhance the overall utility of findings. Due to limited study availability, subgroup analysis for modified tools was unfeasible. Subsequent studies should incorporate a broader array of tools, ensuring standardization and rigorous testing during the design phase to facilitate result comparability.

The prevalence of oral frailty can be affected by various factors such as the use of multiple medications, drug-induced dry mouth, feeding methods, length of hospitalization, and disease severity. Anticholinergic drugs can lead to dry mouth, impair oral self-cleaning, and raise the risk of oral frailty (33). Prolonged reliance on enteral feeding may increase the vulnerability to oral frailty due to the absence of regular oral chewing and swallowing functions (34, 35). Extended hospital stays can worsen the deterioration of oral health (36). The severity of the disease impacts the patient’s overall health status and indirectly influences the occurrence of oral frailty (37). In the current study, due to the limitations of the data, we are unable to conduct an in-depth analysis of confounding factors. This limitation may have a certain impact on the comprehensiveness and accuracy of the research results. Therefore, we suggest that future research fully consider the potential influence of confounding factors in the design stage and take appropriate adjustment measures during the data analysis process to enhance the reliability and scientific nature of the research results.

### Limitations

Several limitations require consideration. First, the single-rate meta-analysis resulted in significant inter-study heterogeneity, which subgroup analyses did not fully explain. Second, although disease type was associated with prevalence, potential confounders such as medication use were not examined. Third, most included studies originated from mainland China, potentially limiting generalizability. Finally, the exclusion of informally published literature may have missed relevant data.

## Conclusion

The results showed that oral frailty affected 51% of hospitalized patients. Patients with cancer were at the highest risk (62%), whereas patients with renal disease had the lowest risk (39%). Disease type, study design, region, age, and sample size influenced oral frailty prevalence. Therefore, as a preliminary recommendation, healthcare systems may consider developing targeted screening protocols for high-risk populations. Subsequently, the implementation of long-term follow-up studies is warranted to validate the presented prevalence estimates and systematically evaluate the effectiveness of any resulting interventions. Meanwhile, multicenter studies are necessary to ensure the applicability of findings across diverse healthcare settings.

## Data Availability

The original contributions presented in the study are included in the article/Supplementary material, further inquiries can be directed to the corresponding author.
