# Supplementary material for: Systematic review and meta-analysis of oral frailty prevalence among older hospitalized patients
Source: Front Public Health. 2025 Dec 11;13:1681594. doi: 10.3389/fpubh.2025.1681594 (PMC12738335; doi:10.3389/fpubh.2025.1681594)
Supplement: Supplementary file 1 [file Table_1.pdf]

Table S1 Search strategy.

PubMed	Search Details	Results
#1	"oral frailty"[Title/Abstract] OR "oral weakness"[Title/Abstract] OR "oral frail*"[Title/Abstract] OR "oral function"[Title/Abstract]	1,844
#2	"patients"[Title/Abstract] OR "patient*"[Title/Abstract] OR "hospitalize*"[Title/Abstract] OR "inpatients"[Title/Abstract] OR "inpatient*"[Title/Abstract]	9,134,006
#3	#1 AND #2	1,092
Web of Science		
#1	AB = “oral frailty” OR AB = “oral weakness” OR AB = “oral frail*” OR AB = “oral function”	2,204
#2	AB = “patients” OR AB = “patient*” OR AB = “hospitalize*” OR AB = “inpatients” OR AB = “inpatient*”	12,116,613
#3	#1 AND #2	1,274
Embase		
#1	'oral frailty':ab,ti OR 'oral weakness':ab,ti OR 'oral frail*':ab,ti OR 'oral function':ab,ti	1,953
#2	patients:ab,ti OR patient*:ab,ti OR hospitalize*:ab,ti OR inpatients:ab,ti OR inpatient*:ab,ti	13,843,231
#3	#1 AND #2	1,235
Scopus		
#1	(TITLE-ABS-KEY ("oral frailty") OR TITLE-ABS-KEY ("oral weakness") OR TITLE-ABS-KEY ("oral frail*") OR TITLE-ABS-KEY ("oral function") AND TITLE-ABS-KEY (patients) OR TITLE-ABS-KEY (patient*) OR TITLE-ABS-KEY (hospitalize*) OR TITLE-ABS-KEY (inpatients) OR TITLE-ABS-KEY (inpatient*))	1,595
Cochrane library		
#1	("oral frailty"):ti,ab,kw OR ("oral weakness"):ti,ab,kw OR (oral NEXT frail*):ti,ab,kw OR ("oral function"):ti,ab,kw	223
#2	(patients):ti,ab,kw OR (hospitalize*):ti,ab,kw OR (inpatients):ti,ab,kw OR (patient*):ti,ab,kw OR (inpatient*):ti,ab,kw	1,324,708
#3	#1 AND #2	132
CNKI	FT = 口腔衰弱 + 口腔功能减退	148
SinoMed	"口腔衰弱"[摘要:智能] OR "口腔功能减退"[摘要:智能]	91
VIP	R=口腔衰弱 OR 口腔功能减退	266
Wanfang data	主题:(口腔衰弱 OR 口腔功能减退)	743

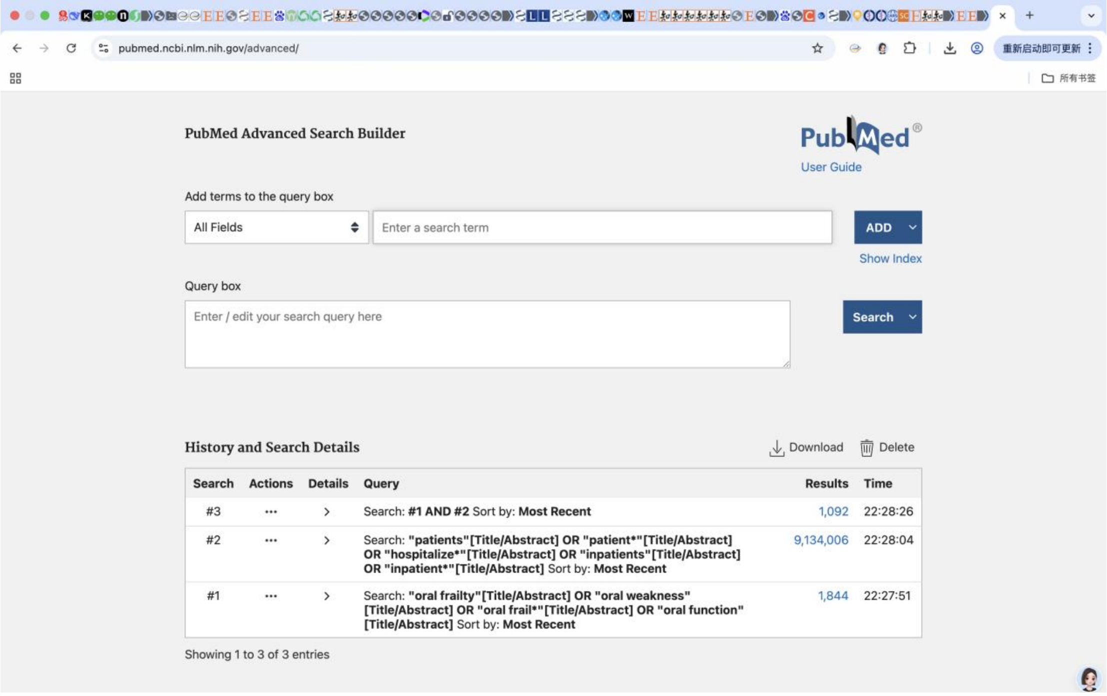
pubmed.ncbi.nlm.nih.gov/advanced/
PubMed Advanced Search Builder
PubMed®
User Guide
Add terms to the query box
All Fields
Enter a search term
ADD
Show Index
Query box
Enter / edit your search query here
Search
History and Search Details
Download
Delete
Search | Actions | Details | Query | Results | Time
#3 ••• > Search: #1 AND #2 Sort by: Most Recent 1,092 22:28:26
#2 ••• > Search: "patients"[Title/Abstract] OR "patient*"[Title/Abstract] OR "hospitalize*"[Title/Abstract] OR "inpatients"[Title/Abstract] OR "inpatient*"[Title/Abstract] Sort by: Most Recent 9,134,006 22:28:04
#1 ••• > Search: "oral frailty"[Title/Abstract] OR "oral weakness" [Title/Abstract] OR "oral frail*"[Title/Abstract] OR "oral function" [Title/Abstract] Sort by: Most Recent 1,844 22:27:51
Showing 1 to 3 of 3 entries

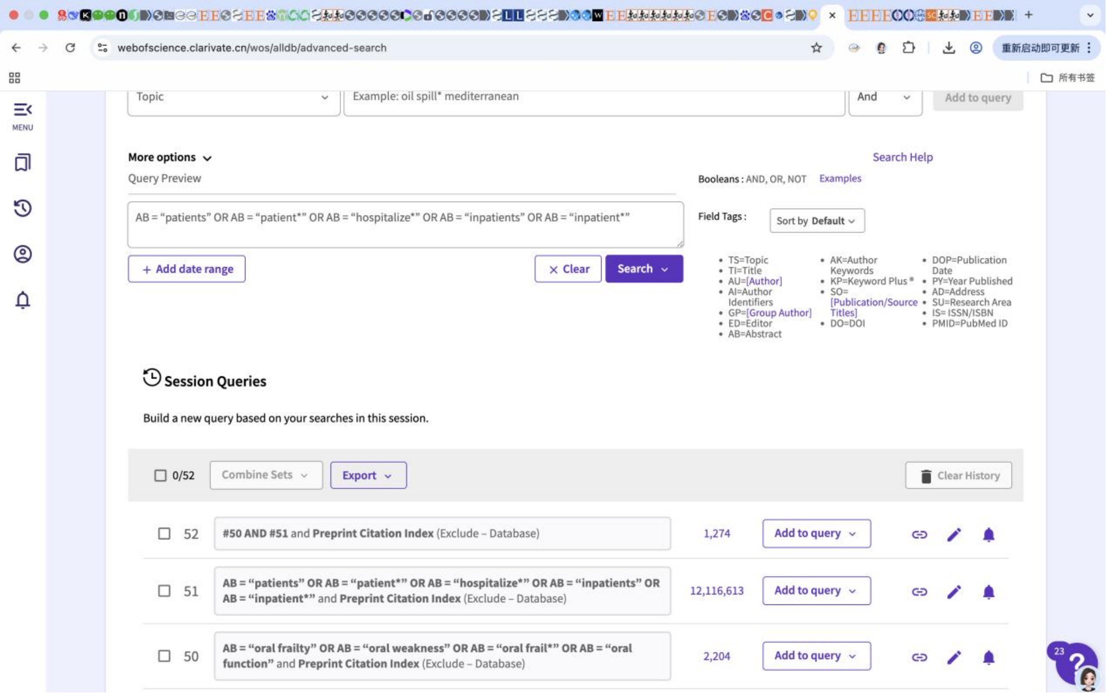
webofscience.clarivate.cn/wos/alldb/advanced-search
Topic
Example: oil spill* mediterranean
And
Add to query
MENU
More options
Search Help
Query Preview
Booleans: AND, OR, NOT Examples
AB = "patients" OR AB = "patient*" OR AB = "hospitalize*" OR AB = "inpatients" OR AB = "inpatient*"
Field Tags:
Sort by Default
+ Add date range
Clear
Search
TS=Topic
TI=Title
AU=[Author]
AI=Author Identifiers
GP=[Group Author]
ED=Editor
AB=Abstract
AK=Author Keywords
KP=Keyword Plus®
SO=[Publication/Source Titles]
DO=DOI
DOP=Publication Date
PY=Year Published
AD=Address
SU=Research Area
IS=ISSN/ISBN
PMID=PubMed ID
Session Queries
Build a new query based on your searches in this session.
0/52
Combine Sets
Export
Clear History
52 #50 AND #51 and Preprint Citation Index (Exclude – Database) 1,274 Add to query
51 AB = "patients" OR AB = "patient*" OR AB = "hospitalize*" OR AB = "inpatients" OR AB = "inpatient*" and Preprint Citation Index (Exclude – Database) 12,116,613 Add to query
50 AB = "oral frailty" OR AB = "oral weakness" OR AB = "oral frail*" OR AB = "oral function" and Preprint Citation Index (Exclude – Database) 2,204 Add to query

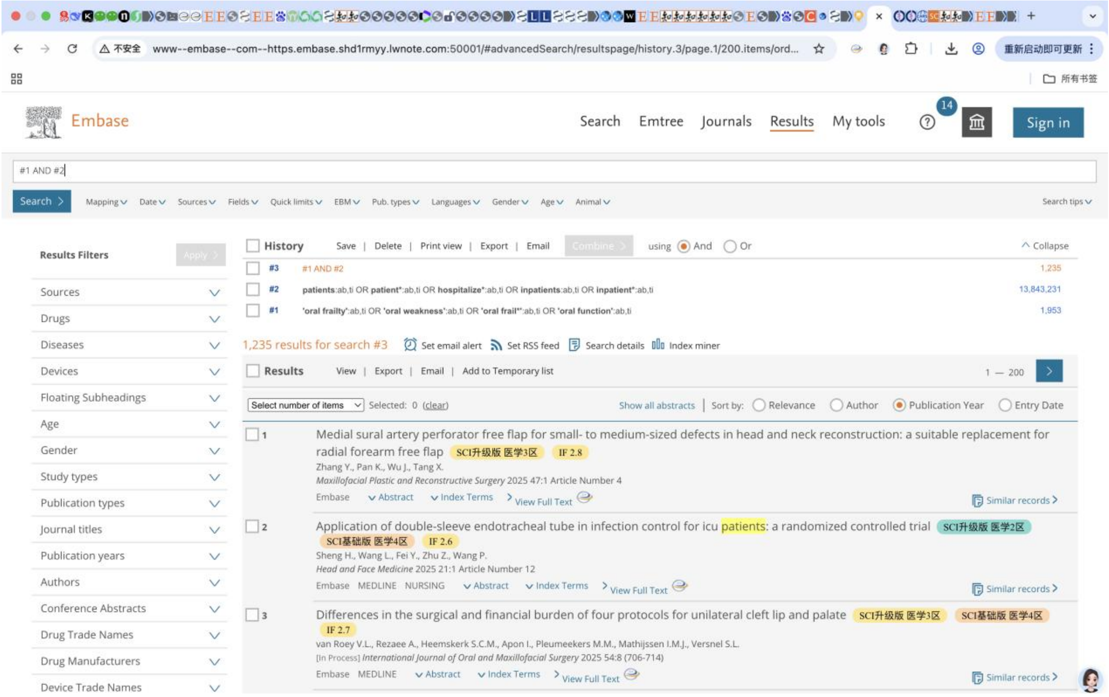
Embase
Search Emtree Journals Results My tools
Sign in
#1 AND #2
Results Filters
Sources
Drugs
Diseases
Devices
Floating Subheadings
Age
Gender
Study types
Publication types
Journal titles
Publication years
Authors
Conference Abstracts
Drug Trade Names
Drug Manufacturers
Device Trade Names
History Save | Delete | Print view | Export | Email Combine using And Or Collapse
#3 #1 AND #2 1,235
#2 patients:ab,ti OR patient*:ab,ti OR hospitalize*:ab,ti OR inpatients:ab,ti OR inpatient*:ab,ti 13,843,231
#1 'oral frailty':ab,ti OR 'oral weakness':ab,ti OR 'oral frail*':ab,ti OR 'oral function':ab,ti 1,953
1,235 results for search #3
Set email alert Set RSS feed Search details Index miner
Results View | Export | Email | Add to Temporary list 1 — 200
Sort by: Relevance Author Publication Year Entry Date
1 Medial sural artery perforator free flap for small- to medium-sized defects in head and neck reconstruction: a suitable replacement for radial forearm free flap SCI升级版 医学3区 IF 2.8
Zhang Y., Pan K., Wu J., Tang X.
Maxillofacial Plastic and Reconstructive Surgery 2025 47:1 Article Number 4
2 Application of double-sleeve endotracheal tube in infection control for icu patients: a randomized controlled trial SCI升级版 医学2区 SCI基础版 医学4区 IF 2.6
Sheng H., Wang L., Fei Y., Zhu Z., Wang P.
Head and Face Medicine 2025 21:1 Article Number 12
3 Differences in the surgical and financial burden of four protocols for unilateral cleft lip and palate SCI升级版 医学3区 SCI基础版 医学4区 IF 2.7
van Roey V.L., Rezaee A., Heemskerk S.C.M., Apon I., Pleumeekers M.M., Mathijssen I.M.J., Versnel S.L.
[In Process] International Journal of Oral and Maxillofacial Surgery 2025 54:8 (706-714)

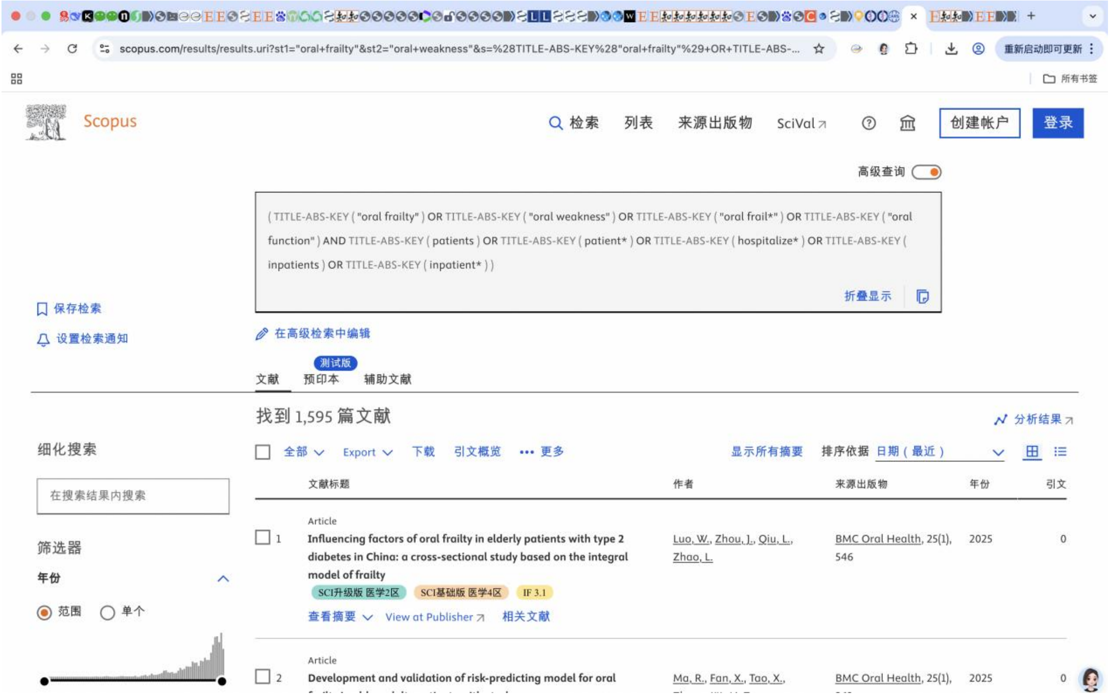
Scopus
检索 列表 来源出版物 SciVal
创建帐户 登录
高级查询
(TITLE-ABS-KEY ("oral frailty") OR TITLE-ABS-KEY ("oral weakness") OR TITLE-ABS-KEY ("oral frail*") OR TITLE-ABS-KEY ("oral function") AND TITLE-ABS-KEY (patients) OR TITLE-ABS-KEY (patient*) OR TITLE-ABS-KEY (hospitalize*) OR TITLE-ABS-KEY (inpatients) OR TITLE-ABS-KEY (inpatient*))
折叠显示
保存检索
设置检索通知
在高级检索中编辑
文献 预印本 辅助文献
找到 1,595 篇文献
分析结果
全部 Export 下载 引文概览 更多
显示所有摘要 排序依据 日期（最近）
文献标题 作者 来源出版物 年份 引文
细化搜索
在搜索结果内搜索
筛选器
年份
范围 单个
Article
1 Influencing factors of oral frailty in elderly patients with type 2 diabetes in China: a cross-sectional study based on the integral model of frailty
Luo, W., Zhou, J., Qiu, L., Zhao, L.
BMC Oral Health, 25(1), 546
2025 0
SCI升级版 医学2区 SCI基础版 医学4区 IF 3.1
查看摘要 View at Publisher 相关文献
Article
2 Development and validation of risk-predicting model for oral
Ma, R., Fan, X., Tao, X.,
BMC Oral Health, 25(1),
2025 0

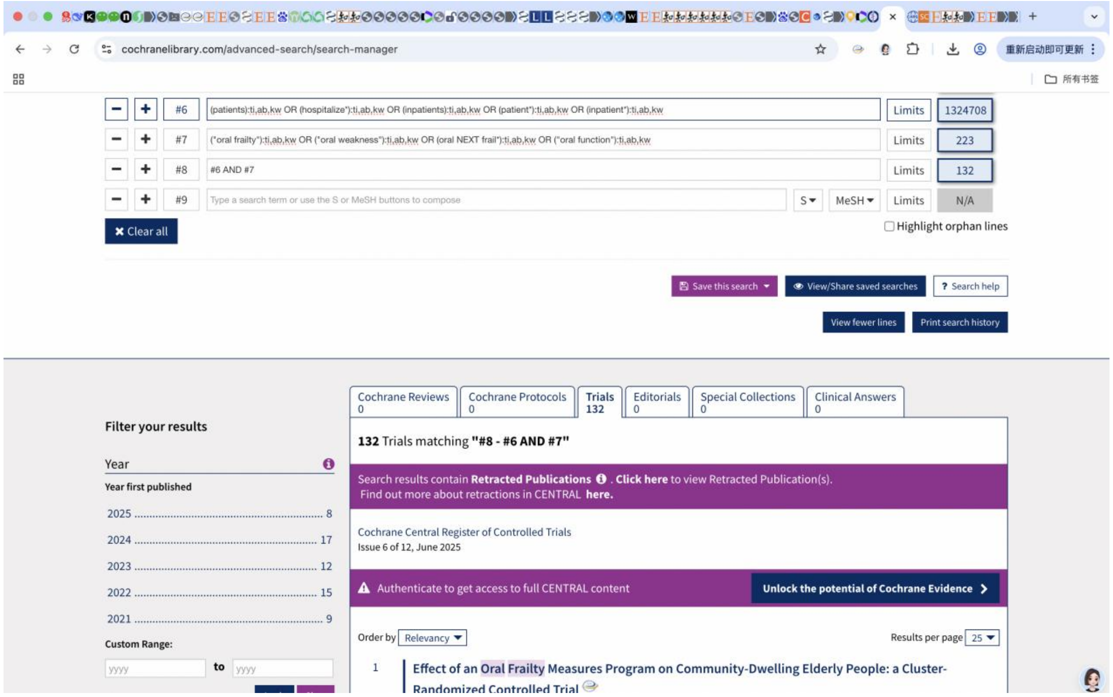
cochranelibrary.com/advanced-search/search-manager
#6 (patients):ti,ab,kw OR (hospitalize*):ti,ab,kw OR (inpatients):ti,ab,kw OR (patient*):ti,ab,kw OR (inpatient*):ti,ab,kw Limits 1324708
#7 ("oral frailty"):ti,ab,kw OR ("oral weakness"):ti,ab,kw OR (oral NEXT frail*):ti,ab,kw OR ("oral function"):ti,ab,kw Limits 223
#8 #6 AND #7 Limits 132
#9 Type a search term or use the S or MeSH buttons to compose S MeSH Limits N/A
Clear all
Highlight orphan lines
Save this search
View/Share saved searches
Search help
View fewer lines
Print search history
Filter your results
Year
Year first published
2025 8
2024 17
2023 12
2022 15
2021 9
Custom Range:
yyyy to yyyy
Cochrane Reviews 0
Cochrane Protocols 0
Trials 132
Editorials 0
Special Collections 0
Clinical Answers 0
132 Trials matching "#8 - #6 AND #7"
Search results contain Retracted Publications. Click here to view Retracted Publication(s).
Find out more about retractions in CENTRAL here.
Cochrane Central Register of Controlled Trials
Issue 6 of 12, June 2025
Authenticate to get access to full CENTRAL content
Unlock the potential of Cochrane Evidence
Order by Relevancy
Results per page 25
1 Effect of an Oral Frailty Measures Program on Community-Dwelling Elderly People: a Cluster-Randomized Controlled Trial

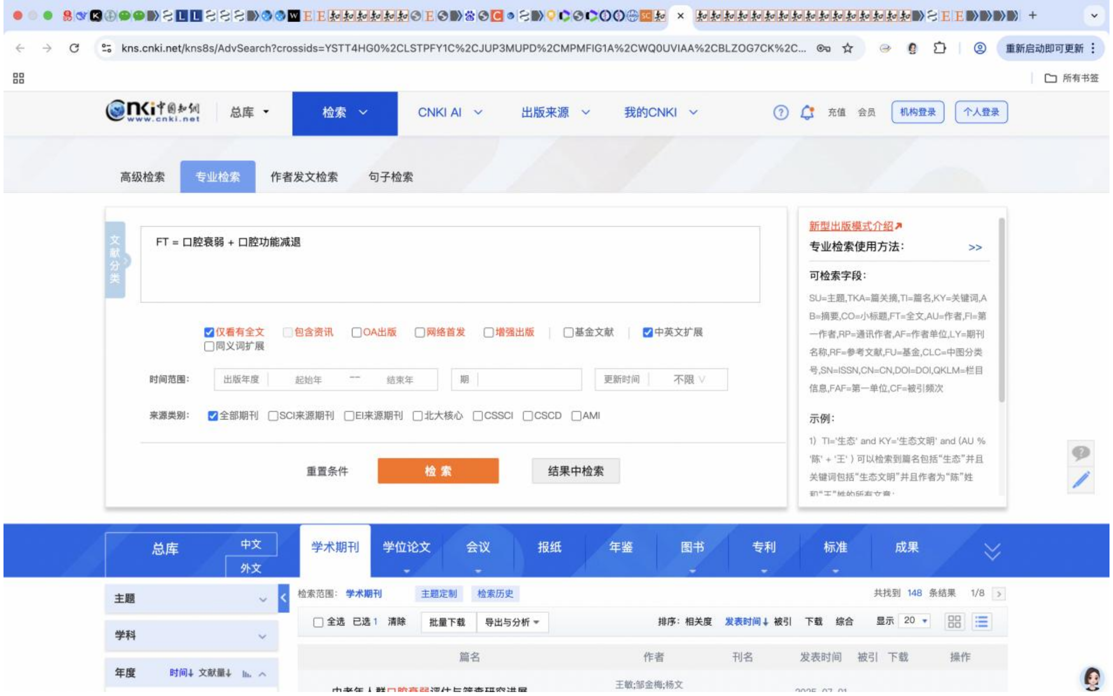
kns.cnki.net/kns8s/AdvSearch?crossids=YSTT4HG0%2CLSTPFY1C%2CJUP3MUPD%2CMPMFIG1A%2CWQ0UVIAA%2CBLZOG7CK%2C...
总库
检索
CNKI AI
出版来源
我的CNKI
充值
会员
机构登录
个人登录
高级检索
专业检索
作者发文检索
句子检索
FT = 口腔衰弱 + 口腔功能减退
仅看有全文
包含资讯
OA出版
网络首发
增强出版
基金文献
中英文扩展
同义词扩展
时间范围:
出版年度
起始年
结束年
更新时间
不限
来源类别:
全部期刊
SCI来源期刊
EI来源期刊
北大核心
CSSCI
CSCD
AMI
重置条件
检 索
结果中检索
新型出版模式介绍
专业检索使用方法:
可检索字段:
SU=主题,TKA=篇关摘,TI=篇名,KY=关键词,AB=摘要,CO=小标题,FT=全文,AU=作者,FI=第一作者,RP=通讯作者,AF=作者单位,LY=期刊名称,RF=参考文献,FU=基金,CLC=中图分类号,SN=ISSN,CN=CN,DOI=DOI,QKLM=栏目信息,FAF=第一单位,CF=被引频次
示例:
学术期刊
学位论文
会议
报纸
年鉴
图书
专利
标准
成果
中文
外文
主题
学科
年度
检索范围: 学术期刊
主题定制
检索历史
共找到 148 条结果
1/8
全选
已选 1
清除
批量下载
导出与分析
排序: 相关度
发表时间
被引
下载
综合
显示 20
篇名
作者
刊名
发表时间
被引
下载
操作

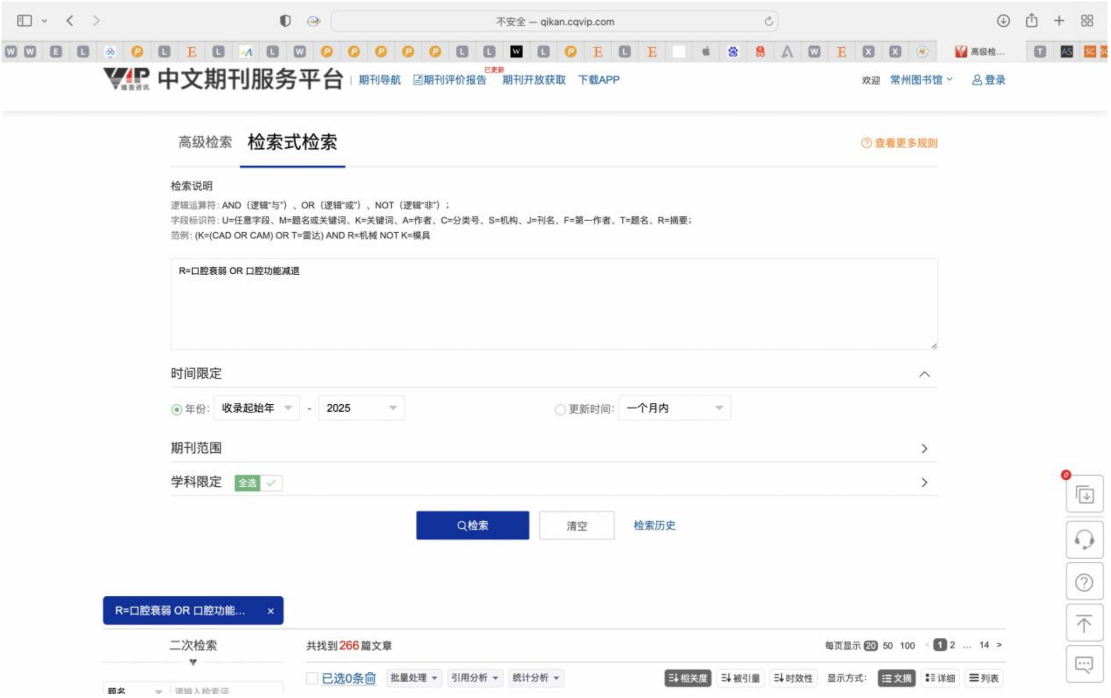
不安全 — qikan.cqvip.com
中文期刊服务平台
期刊导航
期刊评价报告
期刊开放获取
下载APP
欢迎 常州图书馆
登录
高级检索
检索式检索
查看更多规则
检索说明
逻辑运算符：AND（逻辑"与"）、OR（逻辑"或"）、NOT（逻辑"非"）；
字段标识符：U=任意字段、M=题名或关键词、K=关键词、A=作者、C=分类号、S=机构、J=刊名、F=第一作者、T=题名、R=摘要；
范例：(K=(CAD OR CAM) OR T=雷达) AND R=机械 NOT K=模具
R=口腔衰弱 OR 口腔功能减退
时间限定
年份：收录起始年 - 2025
更新时间：一个月内
期刊范围
学科限定 全选
检索
清空
检索历史
R=口腔衰弱 OR 口腔功能...
二次检索
共找到266篇文章
每页显示 20 50 100
已选0条
批量处理
引用分析
统计分析
相关度
被引量
时效性
显示方式：
文摘
详细
列表

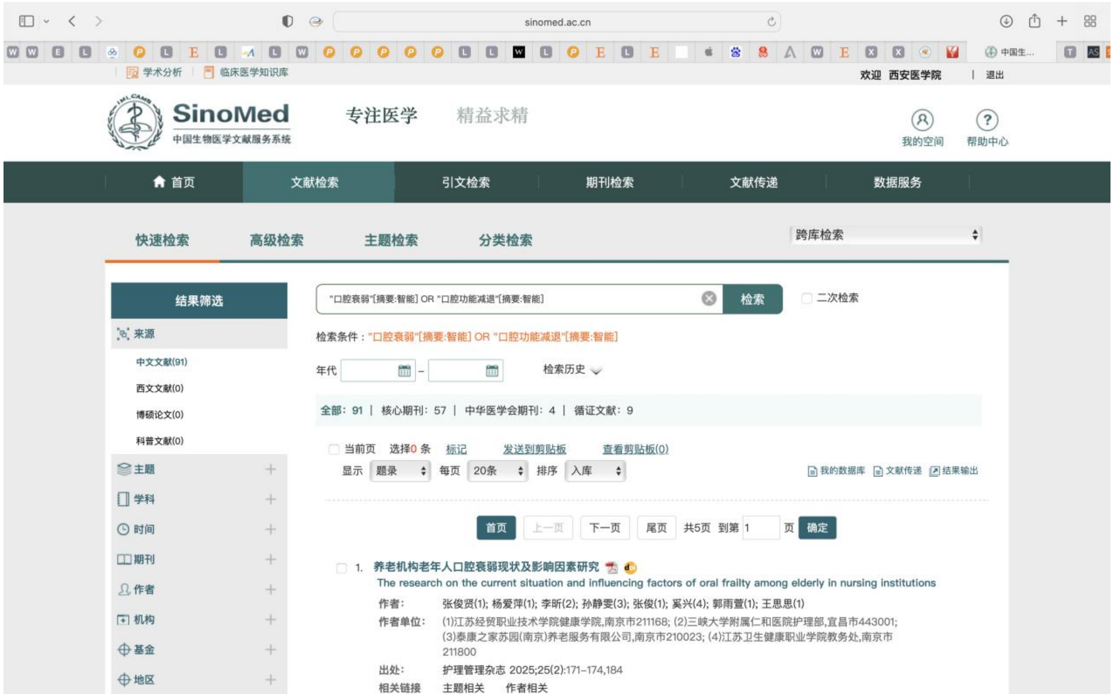
sinomed.ac.cn
学术分析
临床医学知识库
欢迎 西安医学院
退出
SinoMed
中国生物医学文献服务系统
专注医学
精益求精
我的空间
帮助中心
首页
文献检索
引文检索
期刊检索
文献传递
数据服务
快速检索
高级检索
主题检索
分类检索
跨库检索
结果筛选
"口腔衰弱"[摘要:智能] OR "口腔功能减退"[摘要:智能]
检索
二次检索
来源
中文文献(91)
西文文献(0)
博硕论文(0)
科普文献(0)
检索条件："口腔衰弱"[摘要:智能] OR "口腔功能减退"[摘要:智能]
年代
检索历史
全部：91 | 核心期刊：57 | 中华医学会期刊：4 | 循证文献：9
当前页 选择0条 标记 发送到剪贴板 查看剪贴板(0)
显示 题录 每页 20条 排序 入库
我的数据库
文献传递
结果输出
主题
学科
时间
期刊
作者
机构
基金
地区
首页
上一页
下一页
尾页
共5页 到第 1 页 确定
1. 养老机构老年人口腔衰弱现状及影响因素研究
The research on the current situation and influencing factors of oral frailty among elderly in nursing institutions
作者：张俊贤(1); 杨爱萍(1); 李昕(2); 孙静雯(3); 张俊(1); 奚兴(4); 郭雨萱(1); 王思思(1)
作者单位：(1)江苏经贸职业技术学院健康学院,南京市211168; (2)三峡大学附属仁和医院护理部,宜昌市443001; (3)泰康之家苏园(南京)养老服务有限公司,南京市210023; (4)江苏卫生健康职业学院教务处,南京市211800
出处：护理管理杂志 2025;25(2):171-174,184
相关链接 主题相关 作者相关

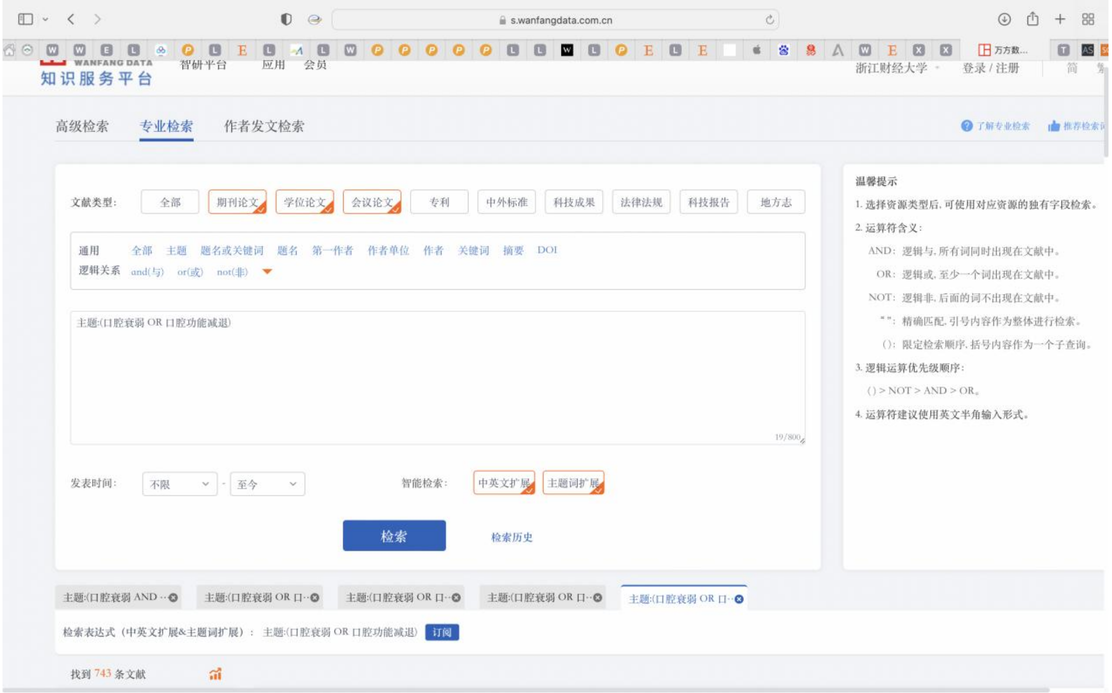
s.wanfangdata.com.cn
WANFANG DATA
知识服务平台
智研平台
应用
会员
浙江财经大学
登录 / 注册
高级检索
专业检索
作者发文检索
了解专业检索
文献类型:
全部
期刊论文
学位论文
会议论文
专利
中外标准
科技成果
法律法规
科技报告
地方志
通用
全部
主题
题名或关键词
题名
第一作者
作者单位
作者
关键词
摘要
DOI
逻辑关系
and(与)
or(或)
not(非)
主题:(口腔衰弱 OR 口腔功能减退)
发表时间:
不限
至今
智能检索:
中英文扩展
主题词扩展
检索
检索历史
温馨提示
1. 选择资源类型后, 可使用对应资源的独有字段检索。
2. 运算符含义:
AND: 逻辑与, 所有词同时出现在文献中。
OR: 逻辑或, 至少一个词出现在文献中。
NOT: 逻辑非, 后面的词不出现在文献中。
" ": 精确匹配, 引号内容作为整体进行检索。
( ): 限定检索顺序, 括号内容作为一个子查询。
3. 逻辑运算优先级顺序:
( ) > NOT > AND > OR。
4. 运算符建议使用英文半角输入形式。
主题:(口腔衰弱 AND ··
主题:(口腔衰弱 OR 口··
主题:(口腔衰弱 OR 口··
主题:(口腔衰弱 OR 口··
主题:(口腔衰弱 OR 口··
检索表达式 (中英文扩展&主题词扩展): 主题:(口腔衰弱 OR 口腔功能减退)
订阅
找到 743 条文献

Table S2
Characteristics of included studies.

Author, Year	Country	Study Design	Diagnosis	Prevalence (%)
Chen et al. 2024[1]	China	Cross-sectional	Maintenance hemodialysis	45.23
Chen et al. 2023[2]	Taiwan	Cross-sectional	Pneumonia	58.30
Chen et al. 2024[3]	Taiwan	Cross-sectional	>3 days of hospitalization	53.40
Miyasato et al. 2024[4]	Japan	Prospective cohort	Hemodialysis	38.80
Xie et al. 2024[5]	China	Cross-sectional	Cerebral small vessel disease	28.57
Yang et al. 2024[6]	China	Cross-sectional	Acutely inpatients	41.27
Dou et al. 2025[7]	China	Cross-sectional	Chronic diseases	48.70
Hu et al. 2024[8]	China	Prospective cohort	Non-cardiac surgery	49.51
Ikuno et al. 2024[9]	Japan	Retrospective cohort	Elective abdominal visceral surgery	43.74
Kobayashi et al. 2025[10]	Japan	Prospective cohort	Peritoneal dialysis	29.41
Li et al. 2025[11]	China	Cross-sectional	Chemotherapy	57.58
Luo et al. 2025[12]	China	Cross-sectional	T2DM	32.95
Ma et al. 2025[13]	China	Cross-sectional	Stroke	47.80
Tian et al. 2025[14]	China	Cross-sectional	T2DM	45.90
Li et al. 2025[15]	China	Cross-sectional	Elderly hospitalized	58.20
Lu et al. 2025[16]	China	Cross-sectional	Cancer	63.50
Wu et al. 2024[17]	China	Cross-sectional	Chronic diseases	30.40
Shao et al. 2025[18]	China	Cross-sectional	Chronic diseases	60.20
Fan et al. 2024[19]	China	Cross-sectional	Stroke	61.50
Shang et al. 2025[20]	China	Cross-sectional	Diabetes	46.82
Hu et al. 2024[21]	China	Cross-sectional	Elderly hospitalized	55.40
Li et al. 2024[22]	China	Cross-sectional	Elderly hospitalized	59.20
Wang et al. 2025[23]	China	Cross-sectional	Chronic diseases	61.43
Shi et al. 2025[24]	China	Cross-sectional	Ischemic stroke	58.18
Li et al. 2024[25]	China	Cross-sectional	Elderly hospitalized	58.50
Li et al. 2024[26]	China	Cross-sectional	Cancer	64.30
Wang et al. 2025[27]	China	Cross-sectional	Stroke	67.70

Note: Abbreviation: OFI-8, Oral Frailty Index-8; T2DM, Type 2 diabetes mellitus

Table S3
Methodological quality appraisal results based on the AHRQ tool for each study.

Study	Item 1	Item 2	Item 3	Item 4	Item 5	Item 6	Item 7	Item 8	Item 9	Item 10	Item 11	Total score	Quality
Chen et al. 2024[1]	Y	Y	Y	Y	N	Y	Y	Y	U	Y	Y	9	H
Chen et al. 2023[2]	Y	Y	Y	Y	N	N	U	Y	U	Y	N	6	M
Chen et al. 2024[3]	Y	Y	Y	Y	N	Y	U	Y	U	Y	U	8	H
Xie et al. 2024[5]	Y	Y	Y	Y	N	U	Y	Y	U	Y	U	8	H
Yang et al. 2024[6]	Y	Y	Y	Y	N	U	Y	Y	Y	Y	N	8	H
Dou et al. 2025[7]	Y	Y	Y	Y	U	N	Y	Y	U	Y	N	7	M
Li et al. 2025[11]	Y	Y	Y	Y	N	U	Y	Y	U	Y	Y	9	H
Luo et al. 2025[12]	Y	Y	Y	Y	U	Y	Y	Y	Y	Y	Y	10	H
Ma et al. 2025[13]	Y	Y	Y	Y	N	N	Y	Y	Y	Y	Y	9	H
Tian et al. 2025[14]	Y	Y	Y	Y	N	Y	Y	Y	U	Y	N	8	H
Li et al. 2025[15]	Y	Y	Y	Y	N	U	U	Y	U	Y	N	7	M
Lu et al. 2025[16]	Y	Y	Y	Y	N	N	Y	Y	U	Y	N	7	M
Wu et al. 2024[17]	Y	Y	Y	Y	N	N	U	Y	U	Y	N	7	M
Shao et al. 2025[18]	Y	Y	Y	Y	N	N	U	Y	N	Y	N	7	M
Fan et al. 2024[19]	Y	Y	Y	Y	N	U	Y	Y	U	Y	N	7	M
Shang et al. 2025[20]	Y	Y	Y	Y	U	Y	Y	Y	U	Y	N	7	M
Hu et al. 2024[21]	Y	Y	Y	Y	N	N	Y	Y	Y	Y	N	8	H
Li et al. 2024[22]	Y	Y	Y	Y	N	Y	Y	Y	U	Y	N	7	M
Wang et al. 2025[23]	Y	Y	Y	Y	N	U	Y	Y	Y	Y	N	9	H
Shi et al. 2025[24]	Y	Y	Y	Y	N	Y	Y	Y	U	Y	Y	9	M
Li et al. 2024[25]	Y	Y	Y	Y	N	Y	Y	Y	N	Y	N	7	M
Li et al. 2024[26]	Y	Y	Y	Y	N	Y	Y	Y	Y	Y	Y	10	H
Wang et al. 2025[27]	Y	Y	Y	Y	N	Y	Y	Y	Y	Y	U	9	H

Note: Y, yes; N, no; U, unclear; H, high quality; M, medium quality.

Item 1: Define the source of information (survey, record review).

Item 2: List inclusion and exclusion criteria for exposed and unexposed subjects (cases and controls) or refer to previous publications.

Item 3: Indicate time period used for identifying patients.

Item 4: Indicate whether or not subjects were consecutive if not population-based.

Item 5: Indicate if evaluators of subjective components of study were masked to other aspects of the status of the participants.

Item 6: Describe any assessments undertaken for quality assurance purposes (e.g., test/retest of primary outcome measurements).

Item 7: Explain any patient exclusions from analysis.

Item 8: Describe how confounding was assessed and/or controlled.

Item 9: If applicable, explain how missing data were handled in the analysis.

Item 10: Summarize patient response rates and completeness of data collection.

Item 11: Clarify what follow-up, if any, was expected and the percentage of patients for which incomplete data or follow-up was obtained

Table S4

Methodological quality appraisal results based on the NOS tool for each study.

Study	Selection				Comparability	Outcome			Total	Quality
	Item 1	Item 2	Item 3	Item 4	Item 5	Item 6	Item 7	Item 8		
Miyasato et al. 2024[4]	1	1	1	1	2	1	1	1	9	H
Hu et al. 2024[8]	1	1	1	1	2	1	1	1	10	H
Ikuno et al. 2024[9]	1	1	1	1	2	1	1	1	9	H
Kobayashi et al. 2025[10]	1	1	1	1	1	1	1	1	8	H

Note: H, high quality; M, medium quality.

Item 1: Representativeness of the exposed cohort.

Item 2: Selection of the non exposed cohort.

Item 3: Ascertainment of exposure.

Item 4: Demonstration that outcome of interest was not present at start of study.

Item 5: Comparability of cohorts on the basis of the design or analysis.

Item 6: Assessment of outcome.

Item 7: Was follow-up long enough for outcomes to occur.

Item 8: Adequacy of follow up of cohorts.

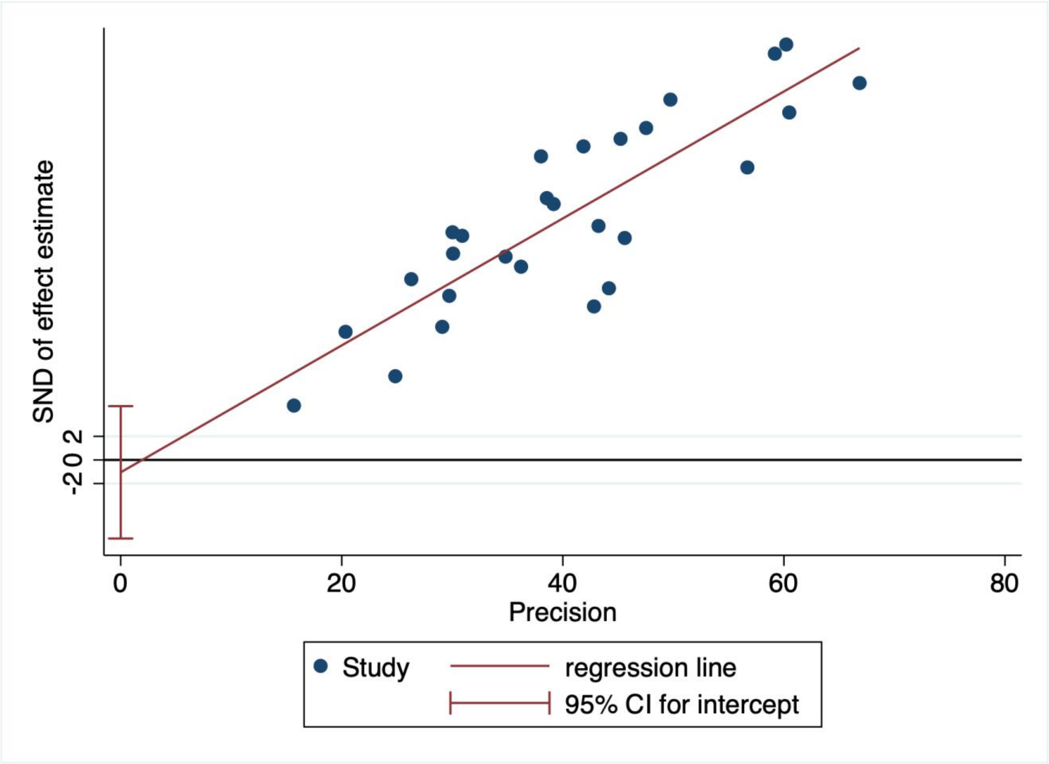

Figure S1. Egger test.

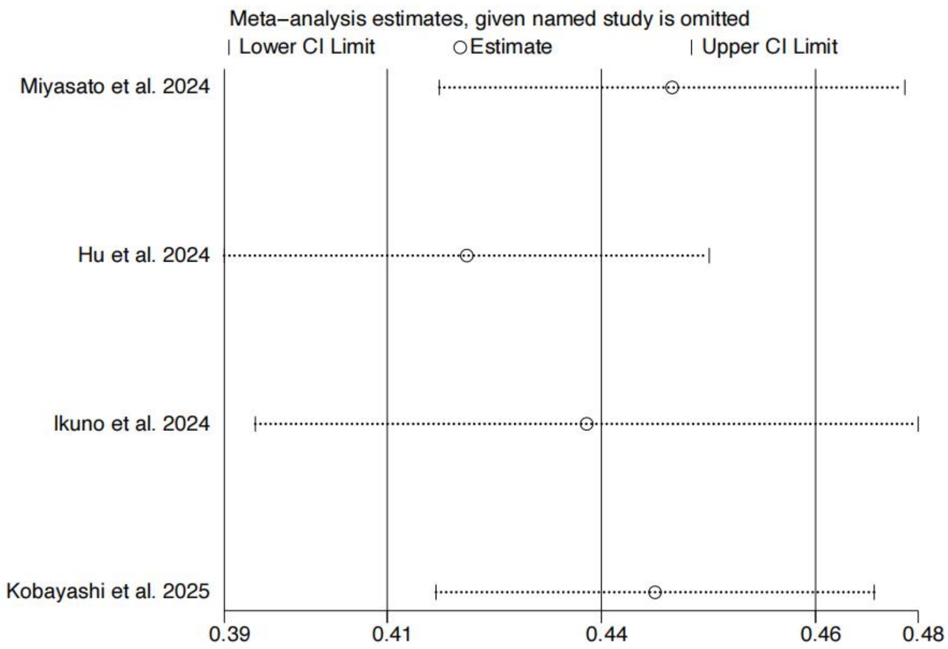

Figure S2. Sensitivity analysis of cohort studies.

References

1. Chen M, He M, Gu Q, Gao X, Lu G. The current status and influencing factors of oral frailty in elderly maintenance hemodialysis patients based on the Andersen Oral Health Outcome Model. BMC Oral Health. (2024)24. doi:10.1186/s12903-024-04872-9.

2. Chen YC, Ku EN, Lin CW, Tsai PF, Wang JL, Yen YF, et al. Tongue pressure during swallowing is an independent risk factor for aspiration pneumonia in middle-aged and older hospitalized patients: An observational study. Geriatrics and Gerontology International. (2023)24:351-357. doi:10.1111/ggi.14769.

3. Chen YC, Ku EN, Tsai PF, Lin CW, Ko NY, Huang ST, et al. The relationship between oral frailty and oral dysbiosis among hospitalized patients aged older than 50 years. Clinical and Experimental Dental Research. (2024)10. doi:10.1002/cre2.890.

4. Miyasato K, Kobayashi Y, Ichijo K, Yamaguchi R, Takashima H, Maruyama T, et al. Oral Frailty as a Risk Factor for Malnutrition and Sarcopenia in Patients on Hemodialysis: A Prospective Cohort Study. Nutrients. (2024)16. doi:10.3390/nu16203467.

5. Xie HY, Chen JL, Xia CQ, Zhang N, Xia ZX, Zhao HY, et al. Association of oral frailty and gait characteristics in patients with cerebral small vessel disease. BMC Neurology. (2024)24. doi:10.1186/s12883-024-03848-0.

6. Yang H, Chen L, Ye D, Wu Y, Zhang H. Oral Health and Its Associated Factors Among the Elderly in the Emergency Department: A Latent Class Analysis. Journal of Multidisciplinary Healthcare. (2024)17:3141-3153. doi:10.2147/JMDH.S469086.

7. Dou JK, Liu H, Min J, Luo Y, Liu Q, Shi XZ, et al.Zhang M. Prevalence and associated factors with oral frailty in middle-aged and older hospitalized patients: a cross-sectional study. Frontiers in Public Health. (2025)13. doi:10.3389/fpubh.2025.1446862.

8. Hu XY, Duan HW, Wang LY, Liu QF, Yao H, Ma DQ, et al. Associations between oral frailty, oral microbiota composition, and postoperative delirium in older adult patients. Journal of the American Geriatrics Society. (2025)73:812-823. doi:10.1111/jgs.19315.

9. Ikuno T, Ida M, Momota Y, Kawaguchi M. Effect of Oral Frailty on Postoperative Infection in Patients Undergoing Abdominal Visceral Surgery. Oral Diseases. (2025)31:633-639. doi:10.1111/odi.15162.

10. Kobayashi Y, Matsuoka T, Yamaguchi R, Ichijo K, Suzuki M, Saito T, et al. Association of Oral Frailty with Physical Frailty and Malnutrition in Patients on Peritoneal Dialysis. Nutrients. (2025)17. doi:10.3390/nu17121950.

11. Li F, Xiao T, Qiu X, Liu C, Ma Q, Yu D, et al. Oral frailty and its influencing factors in patients with cancer undergoing chemotherapy: a cross-sectional study. BMC Oral Health. (2025)25. doi:10.1186/s12903-025-05789-7.

12. Luo W, Zhou J, Qiu L, Zhao L. Influencing factors of oral frailty in elderly patients with type 2 diabetes in China: a cross-sectional study based on the integral model of frailty. BMC Oral Health. (2025)25. doi:10.1186/s12903-025-05815-8.

13. Ma R, Fan X, Tao X, Zhang W, Li Z. Development and validation of risk-predicting model for oral frailty in older adults patients with stroke. BMC Oral Health. (2025)25. doi:10.1186/s12903-025-05428-1.

14. Tian C, Li N, Gao Y, Yan Y. Analysis of the current status and influencing factors of oral frailty in elderly patients with type 2 diabetes mellitus in Taiyuan, China. BMC Geriatrics. (2025)25. doi:10.1186/s12877-025-06052-y.

15. Li ZY, Pan YW, Zhou HL, Zhou HR, Li YZ. Chain mediating effect of social support and nutrition on the relationship between oral frailty and general frailty in elderly hospitalized patients. Journal of Wannan Medical College. (2025)44.

16. Lu CQ, Lu QY, Jin XQ, Song J. Construction and validation of a risk prediction model for oral frailty in elderly cancer patients. Chinese Journal of Geriatric Dentistry. (2025)23.

17. Wu YR, Jiao M, Zhu M, Han YT, Tao XB, Wang XY. Current situation and influencing factors of oral frailty in the elderly hospitalized patients with chronic diseases. Journal of MuDanJiang Medical University. (2024)45.

18. Shao M, Chi CR, Huang XH, Chen MQ, Yang D, Yuan T, et al. Current Situation of Occurrence and Influencing Factors of Oral Frailty in the Elderly Inpatients with Chronic Diseases. Journal of Changzhi Medical College. (2025)39.

19. Fan XL, Ma RR, Zhang W, Liu YJ. Current status and influencing factors analysis of oraI frailty in elderly stroke patient. Journal of Qiqihar Medical University. (2024)45.

20. Shang XH, Du YF, Wen BL, Jia QM, Zheng Y, Hu YN, et al. Current status and influencing factors of oral frailty in elderly diabetic patients. Chinese Journal of Modern Nursing. (2025)31.

21. Hu HM, Li C, Hu KK, Chang AR, Lu JW, Huang GL, et al. Status and influencing factors of oral frailty in elderly inpatients. Chinese Evidence-Based Nursing. (2024)10.

22. Li ZY, Pan YW, Zhou HL, Wei ZD, Li YZ. Construction and validation of a risk prediction model for oral frailty in elderly in patients. Journal of Mudanjiang Medical College. (2024)45.

23. Wang XS, Ye LX, Chen L, Wang XY, Cai XX. Analysis of the current situation and influencing factors of oral frailty in chronic disease inpatients. Chronic Pathematology Journal. (2025)26.

24. Shi Y, Yu HL, Gu ZE, Huang HW, Ji L, Wang L, et al. Trajectory and influencing factors of oral frailty in first-onset elderly patients with ischemic stroke. Chinese Journal of Modern Nursing. (2025)31.

25. Li ZY, Pan YW, Zhou HL, Zhou HR, Li YZ, Kuang X. Current Situation of Oral Frailty in Elderly Inpatients in Wuhu City and Analysis of Its Influencing Factors. Journal of Qiqihar Medical University. (2024)45.

26. Li Y, Zhang ZY, Zou YL, Li X, Yang LH, Xia C, et al. Oral frailty and its influencing factors among hospitalized cancer patients. Journal of Nursing Science. (2024)39.

27. Wang T, Li DJ, Li CB, Peng SJ, Chen MM, Dong QS, et al. Epidemiological Characteristics and Influencing Factors of Oral Frailty in Hospitalized Stroke Patients: an Observational Study. Practical Journal of Cardiac Cerebral Pneumal and Vascular Disease. (2025)33.
